# Co-infection with Pansensitive and Multidrug-Resistant Strains of *Mycobacterium tuberculosis*

**DOI:** 10.3201/eid1504.080592

**Published:** 2009-04

**Authors:** Michael P. Mendez, Mary E. Landon, Mary K. McCloud, Peter Davidson, Paul J. Christensen

**Affiliations:** Department of Veterans Affairs Medical Center, Ann Arbor, Michigan, USA (M.P. Mendez, P.J. Christensen); Washtenzw County Public Health Department, Ypsilanti, Michigan, USA (M.E. Landon M.K. McCloud); Department of Community Health, Lansing, Michigan, USA (P. Davidson)

**Keywords:** Antimicrobial resistance, tuberculosis, Mycobacteria, Mycobacterium tuberculosis, spoligotyping, dispatch

## Abstract

We report a case of a 23-year-old HIV-negative man with multidrug-resistant *Mycobacterium tuberculosis* that became evident while he was being treated for *M. tuberculosis* that was sensitive to all first-line drugs. This case should alert clinicians to consider co-infection as a possible cause of recrudescent disease.

A 23-year-old HIV-negative man from Somalia immigrated to the United States 1.5 years before seeking treatment for his symptoms. The patient had a 3-week history of dry cough, dyspnea with exertion, pleuritic chest pain, fatigue, weight loss, and night sweats. A chest radiograph showed extensive left hemithorax opacification, a left apical cavitary lesion, and right apical nodular lesions (Figure, panel A). Acid-fast bacillus (AFB) smears and culture were positive for *Mycobacterium tuberculosis*. Drug-susceptibility testing demonstrated sensitivity to all first-line drugs. He received directly observed therapy (DOT), which consisted of isoniazid (300 mg/d), pyrazinamide (1,000 mg/d), rifampin (600 mg/d), and ethambutol (1,200 mg/d).

**Figure:**
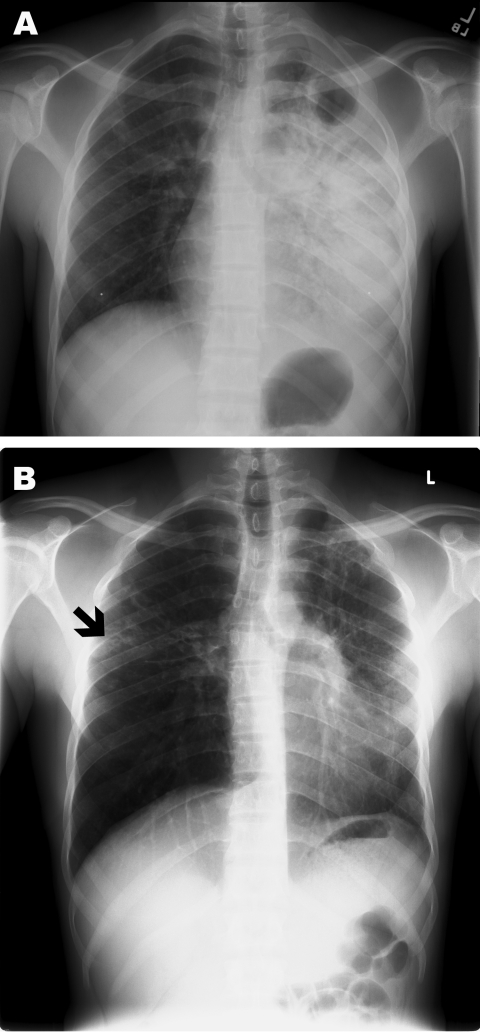
Poster-anterior chest radiographs of patient with multidrug-resistant tuberculosis. A) Radiograph taken at diagnosis, demonstrating dense consolidation of the left lower lobe and lingula. A left apical cavity is present. Minimal change is also noted in the right mid-lung zone. B) Radiograph taken after 5 months of directly observed therapy. Marked clearance is noted on the left; however, a new small cavitary lesion with surrounding infiltrate is noted in the right mid-lung zone (black arrow).

One month after starting treatment, he had persistent fatigue and shortness of breath and had gained no weight; follow-up chest radiograph showed no change. As a result, he received a prednisone dosage of 40 mg/d with a tapered dose to no prednisone over 2 months (*1**,**2*). Thereafter, he improved rapidly both symptomatically and radiographically. His cough abated, and he was unable to produce sputum.

After 5 months of DOT, he again lost weight and felt fatigued. A repeat chest radiograph showed a new small cavitary lesion in the middle lobe of the right lung (Figure, panel B). At that time, repeat sputum sample was smear negative, but culture was positive for multidrug-resistant tuberculosis (MDR TB). Molecular characterization by spoligotyping and mycobacterial interspersed repeat units showed that the isolate from episode 1 differed genetically from that of episode 2 (Table) (*3**,**4*). Review of laboratory records demonstrated that neither a switch in specimens nor cross-contamination were causes of this new finding. Repeat HIV testing was negative. Careful review of the patient’s contacts, exposures, and travel history did not show any obvious source of reinfection. The patient did not travel outside of the county before or after initial treatment.

**Table:** Selected analyses of sputum from patient with tuberculosis over the course of treatment during 2007*

Date	Isolate	Smear	Culture	Resistance†	Spoligotype	MIRU
Jan 4	1	Positive	Positive	None	000000004020771	224323-53313
Jul 12	2	Negative	Positive	I, R, P, E	777756777760771	224325153226
Sep 8	2	Positive	Positive	I, R, P, E	777756777760771	224325153226

*Molecular characterization was performed on Sep 8 to confirm the presence of the second isolate and reassess for presence of the original isolate. MIRU, mycobacterial interspersed repeat units. †I, isoniazid; R, rifampin; P, pyrazinamide; E, ethambutol.

The patient received amikacin 15 mg/kg/d intravenously, moxifloxicin 400 mg/d, cycloserine 750 mg/d, and aminosalicylic acid 8 gm/d in accordance with drug-susceptibility testing. Because of the extent of previous disease, surgical removal of the diseased lung was not performed. He was kept in isolation until 3 sputum smears were AFB negative. He gained weight, his energy increased, and radiographic appearance of his lungs improved. At the time of his discharge, dosage of amikacin was decreased to 3×/wk, and cycloserine was decreased to 250 mg/d based on the serum levels of each drug. The patient was instructed to receive treatment for a minimum of 24 months after the first negative sputum culture.

Molecular characterization showed that the 2 strains of *M. tuberculosis* were not related (*3**,**4*). Thus, the cause of treatment failure most likely was not due to development of resistance by the infecting strain. Among the >35,000 *M. tuberculosis* isolates characterized by the Centers for Disease Control and Prevention’s National Tuberculosis Genotyping Service (*5*), no exact genotype matches were found for the isolate from the first episode and 3 exact genotype matches were found for the isolate from the second episode (P. Moonan, pers. comm.). All 3 matched genotypes came from persons originating from the same African region (Somalia and Ethiopia), which suggests a phylogeographic lineage adapted to a particular human population (*6*). However, none of the matches clustered in the same US region as the current case. Further investigation determined that interstate transmission in the United States was unlikely. A review of risk factors for reinfection, such as travel to areas endemic for TB or exposure to high-risk populations, was negative, suggesting that the second strain was acquired before treatment. On the basis of this information, we speculate that the patient acquired both strains before traveling to the United States.

We report an immunocompetent person with 2 strains of *M. tuberculosis*, one sensitive to all first-line antituberculous drugs and one multidrug resistant. This case demonstrates the ability of genotyping information to identify simultaneous infection with multiple strains of *M. tuberculosis* in a patient in whom treatment failure is suspected. In addition, this case exemplifies the critical importance of clinical monitoring during DOT in the prompt recognition and treatment of patients who have unsuspected simultaneous infections with multiple strains of *M. tuberculosis*.

In patients who are not receiving DOT, a likely cause of treatment failure is nonadherence to the drug regimen (*7*). In patients who are receiving reliable DOT, deterioration may be explained by cryptic nonadherence, malabsorption, or laboratory error (*7*). In addition, exogenous reinfection should be considered as a possible reason for treatment failure or disease relapse (*8**–**11*). However, an underrecognized cause of treatment failure is mixed infection with >2 strains of *M. tuberculosis* (*12**–**15*). As reported previously (*12*), simultaneous infection with 2 competing strains should be considered when other common reasons are ruled out and a high index of suspicion is present. In the current case, the patient was receiving DOT and demonstrated clinical improvement while receiving an appropriate regimen based on drug-susceptibility testing. When his condition deteriorated clinically and sputum samples were culture positive, several possible causes of treatment failure were quickly discarded, including malabsorption, cryptic nonadherence, and laboratory error/contamination.

As has been previously hypothesized, we conclude that this patient originally harbored both strains (*12*). The effect of disease at the patient’s initial presentation appears to have been predominantly due to the drug-sensitive strain, as evidenced by his initial clinical and radiographic improvement and sputum conversion. The successful treatment of the drug-susceptible strain and the inherent resistance of the second strain to the first-line medications enabled disease from the MDR TB strain to become apparent.

The initial therapy in this case was complicated by delayed clinical response. The use of corticosteroids as an adjuvant to antituberculosis medications in selected patients with severe disease has been documented to hasten resolution of symptoms and radiographic recovery without delaying clearance of organisms (*1**,**2*). Although the role of prednisone treatment in the emergence of the second strain is not clear, we believe the most important factor was the inherent antimicrobial resistance pattern of the second strain.

This case demonstrates the utility of DOT in assisting with prompt recognition of a new infecting strain and highlights the use of genotyping in the assessment of possible treatment failure. Heightened awareness of possible infection with multiple strains, either from reinfection or coinfection, is critical when monitoring patients who are not improving or who recrudesce despite therapy guided by drug-resistance testing. The use of genotyping is a necessary tool in the evaluation of these patients. In particular, as in this case, a high index of suspicion for co-infection should be applied to persons originating from areas in which *M. tuberculosis* is hyperendemic.
